# Assessment of healthcare access and knowledge of HIV pre-exposure prophylaxis in women engaging in sex work: An exploratory study in Lille, Northern France

**DOI:** 10.1097/MD.0000000000049609

**Published:** 2026-07-24

**Authors:** Jérémie Robinsohn, Agnès Meybeck, Emmanuelle Bontemps, Macha Tetart, Michel Valette, Olivier Robineau

**Affiliations:** a Departement of Infectious Diseases, Dron Hospital, Tourcoing, France; b Free Centre for Information, Screening and Diagnosis of STI, Tourcoing, France.

**Keywords:** HIV pre-exposure prophylaxis, sex worker, women

## Abstract

In the context of the extension of HIV pre-exposure prophylaxis (PrEP) prescription to all physicians in France, we conducted an exploratory study investigating care access and PrEP knowledge in women engaging in sex work (WSW) in Lille, Northern France. A cross-sectional quantitative study was conducted from May to September 2024 through a questionnaire administered to all WSW who attended the meetings of “Le Mouvement du Nid,” a nonprofit association supporting WSW. Sociodemographic characteristics, healthcare uptake, sexual practices, PrEP knowledge, and use were assessed according to self-reported data via a structured questionnaire. Forty-five WSW were included: 42 cisgender WSW, 3 transgender WSW. The median age was 36 years. All WSW but 2 were migrants, mainly from sub-Saharan Africa (87%). Vulnerability factors were frequent, especially difficulties with access to social rights (58%) or housing (67%). WSW were often victims of violence (67%). The majority of WSW (76%) reported having a family doctor, and 42% consulting 1 within a year. Only 22% had disclosed engaging in sex work to their family doctor. Screening for sexually transmitted infections has been done by 64% of WSW in the last year, rarely with their family doctor (18%). Four (9%) WSW reported a history of postexposure prophylaxis, all were cisgender WSW. Four (9%) WSW knew about PrEP, including 1 cisgender WSW. No WSW was using PrEP. After receiving education about PrEP, 51% of WSW were interested with PrEP. Knowledge of HIV prevention tools was poor among WSW. Enhancement of sexual healthcare of WSW among family doctors may help PrEP implementation.

## 1. Introduction

HIV pre-exposure prevention (PrEP) involves taking antiretroviral drugs to prevent HIV infection. Women engaging in sex work (WSW) are at significant risk of contracting sexually transmitted infections (STI), including HIV infection, because of sexual risk behaviors, structural vulnerabilities such as stigma, criminalization, economic insecurity, and limited access to healthcare.^[1,2]^

In France, where PrEP is fully reimbursed under the national health system, WSW has been recognized as a population at high risk of acquiring HIV and a key population for PrEP implementation since it became nationally available.^[3]^

In practice, 42,159 people were under PrEP in France in 2021, the vast majority of whom were men.^[4]^ The proportion of women under PrEP slightly increased, rising from 2% in the first half of 2021 to 4% in the second half of 2022. Taking into account that already in 2014, the Central Office for the Suppression of Human Trafficking (OCRTEH) estimated the number of WSW at 25,500, this at-risk population is clearly underrepresented among PrEP users.^[5]^

Initially, the introduction of PrEP in France was restricted to hospital doctors or those working in STI prevention centers. The EPI PHARE study, by highlighting a clear decrease in the prescription of PrEP in the context of the coronavirus disease 2019 pandemic, urged the French Health Authorities to authorize the prescription of PrEP by all practitioners as of June 1, 2021.^[4,6]^

In the context of the extension of the authorization for PrEP prescription, we conducted a study to evaluate access to care, knowledge, and use of PrEP among WSW in Lille, Northern France.

## 2. Patients and methods

### 2.1. Study design

We conducted a cross-sectional survey via a structured questionnaire. From May to September 2024, a questionnaire was administered to WSW who attended the meetings of the association “Le Mouvement du Nid” in Lille, a nonprofit French association supporting sex workers. The association reports meeting more than 3500 WSW throughout France each year thanks to the actions of 25 different delegations. In Lille, it is estimated that around 200 WSW attend support meetings each year. We planned to meet 80 WSW during the study period, hoping to include 50 WSW in the study. WSW were invited to participate if they were fluent in spoken French, English, or Spanish and had a literacy level sufficient to understand the nonopposition note and were willing to answer the questionnaire with the help of the study staff.

### 2.2. Ethic

This observational study was based exclusively on questionnaires and involved no medical or biological intervention and no modification of participants’ care. According to the applicable French regulatory framework, ethics committee approval was not required. Given the vulnerability of the study population and the potential risks of stigmatization and loss of anonymity, a nonopposition procedure was used. Participants received written information, supplemented by oral explanations when necessary, and were free to decline participation. No directly or indirectly identifiable personal data were collected. Data were collected anonymously and handled in accordance with applicable French data protection requirements and National Data Processing and Freedoms Commission (CNIL) regulations.

### 2.3. Data collection

All data were collected via a self-administered questionnaire and were anonymous. Sociodemographic characteristics and sexual practices were collected. An English translation of the questionnaire is available as File S1, Supplemental Digital Content 1. Sexual risk behaviors were assessed by the number of daily clients, the frequency of condom use, and the violence suffered. Self-reported use of condoms was rated from never to always during penetrative sex and oral sex. Verbal, physical, and sexual violence were investigated, as well as perpetrators (client, partner…). Substance use, including tobacco, cannabis, cocaine, heroin, and alcohol was categorized as currently used yes or no. The state of health and recourse to healthcare were questioned in particular, screening for STIs and the use of postexposure prophylaxis (PEP). Finally, the knowledge regarding PrEP and its possible use was informed.

### 2.4. Statistics

Categorical variables are expressed in terms of frequency and percentage. Continuous variables were expressed as mean values ± standard deviation or as median (interquartile range), depending on the normality of their distribution.

## 3. Results

### 3.1. Sociodemographic characteristics and sexual risk behaviors

A total of 45 WSW completed the questionnaire: 42 cisgender women and 3 transgender women. Table 1 summarizes the sociodemographic and sexual risk behaviors data. The median age was 36 years. All WSW but 2 were migrants, mainly from sub-Saharan Africa (87%). Vulnerability factors were frequent, especially difficulties with access to social rights (58%) or housing (67%). Most of the participants had a secondary or lower level of education (64%).

**Table 1:** Sociodemographic characteristics and sexual risk behaviors.

Characteristics	Women engaging in sex work
	(n = 45)
Sociodemographic characteristics	
Age (years)	36 (IQR: 13)
Sex	
Cisgender women	42 (93%)
Transgender women	3 (7%)
Country of birth	
Sub-Saharan Africa	39 (87%)
South-America	4 (9%)
France	2 (4%)
Way of sex work	
Independant	23 (51%)
Network	18 (40%)
Internet	4 (9%)
Parental status	
Without a child	17 (38%)
One child	13 (29%)
≥2 Children	15 (33%)
Social environment	
Isolate	26 (58%)
Friends	14 (31%)
Family	5 (11%)
In a relationship	2 (4%)
Accomodation	
In a home	17 (38%)
Homeless	4 (9%)
At a friend’s house	9 (20%)
In (co)rental	13 (29%)
Education	
None	3 (7%)
Primary school	9 (20%)
Secondary school	17 (38%)
Graduate	10 (22%)
University	6 (13%)
Health coverage	
No health insurance	8 (18%)
Emergency health coverage without	18 (40%)
Sexual risk behaviors	
Number of daily clients	
0–2	13 (29%)
2–4	18 (40%)
5–10	10 (22%)
>10	2 (4%)
Did not wish to answer	2 (4%)
Use of condoms	
Never	1 (2%)
Sometimes	0
Often	4 (9%)
Almost always	7 (16%)
Always	33 (73%)
Violences	30 (67%)
Physical	11 (24%)
Sexual	12 (27%)
Verbal	20 (44%)
Forced sexual intercourse without a condom	15 (33%)

IQR = interquartile range.

Half of the WSW surveyed (51%) declared that they operated independently; 40% belonged to a prostitution network. A minority exercised via the internet (7%). Twenty-eight participants (62%) declared a number of daily clients ≥2. WSW were often victims of violence (67%). The majority of WSW declared using condoms frequently: always or almost always (89%). However, 33% had been forced to have sex without condoms. The use of condoms dropped when oral sex was specifically questioned: always or almost always (68%).

### 3.2. State of health and use of care

Table 2 summarizes the state of health and recourse to healthcare. Nineteen WSW (42%) reported good or very good health. The most frequent chronic diseases were psychological issues (31%) and hypertension (20%). One participant was HIV positive; 3 had chronic hepatitis B. Substance use was reported by 31% of WSW, mainly alcohol. The use of psychotropic drugs was frequent.

**Table 2:** State of health and use of care.

Characteristics	Women sex workers (n = 45)
State of health	
Chronic diseases	
Psychological issues	14 (31%)
Hypertension	9 (20%)
Diabetes	2 (4%)
HIV infection	1 (2%)
Chronic hepatitis B	3 (7%)
Substance use	
Tobacco	5 (11%)
Alcohol	8 (18%)
Cannabis	1 (2%)
Cocaine	2 (4%)
Anxiolytics	15 (33%)
Antidepressants	10 (22%)
Sleeping pills	8 (18%)
Sexual and reproductive health	
Contraception	15/42 (36%)
Contraceptive pills	10 (24%)
Implants	2 (5%)
Intrauterine device	3 (7%)
History of abortion	15/42 (36%)
STI screening in the last 12 months	28 (62%)
In an association	11 (24%)
With a family doctor	8 (18%)
With a gynecologist	4 (9%)
At the emergency department	3 (7%)
Free center for STI screening	0
Ever used PEP	4 (9%)
Knew about PrEP	4 (9%)
Ever used PrEP	0
Intention to use PrEP after information	23 (51%)

PEP = postexposure prophylaxis, PrEP = pre-exposure prophylaxis, STI = sexually transmitted infections.

The majority of respondents (76%) declared having a primary care physician, but only 10 had informed their physician of their professional activity. Thirteen of the WSW interviewed (29%) stated that they had given up on a medical appointment because of the language barrier (n = 2), the lack of a residence permit (n = 4), the fear of stigma (n = 3), or financial issues (n = 4).

### 3.3. Sexual health, PEP, and PrEP

Among cisgender WSW, 15 (36%) had an abortion. Only 15 of the 42 (36%) cisgender WSW who should have been using contraception were using it at the time of the survey. Regarding STIs, 20 participants (44%) declared being well informed (17/42 [40%] cisgender WSW vs 3/3 [100%] transgender WSW; *P* = .08). Twenty-nine (64%) WSW had been tested for STIs during the last year (25/42 [60%] cisgender WSW vs 3/3 [100%] transgender WSW; *P* = .43), by their family doctor for 8 of them. Seven WSW (16%) had been diagnosed with an STI.

Four (9%) WSW reported a history of PEP; all were cisgender women. The main reasons for not using PEP were a lack of awareness of this prevention tool (51%) and the absence of perception of sexual risk-taking (39%).

Four (9%) WSW knew about PrEP (2% of cisgender WSW vs 100% of transgender WSW; *P* < .001). No WSW was using PrEP. After receiving education about PrEP, 51% of WSW declared being interested in PrEP.

## 4. Discussion

This exploratory study showed that WSW in Lille were exposed to significant vulnerabilities and engaged in risky sexual behaviors, confirming the need for various HIV prevention tools, including PEP and PrEP. Access to healthcare was impaired by fear of stigma, lack of social rights, and financial issues. The proportion of WSW with a general practitioner was high. However, the role of the general practitioner in taking care of sexual health in this population was limited. The main determinant of the rare use of PEP by WSW was a lack of knowledge and absence of risk perception. PrEP awareness was very low, and its use was 0. But after the information, the intention to use PrEP was quite high.

Our results revealed targets for future interventions to enhance sexual health and the implementation of PrEP in WSW in Northern France. The main barrier for PrEP use among WSW in Lille is the lack of knowledge of this prevention tool. Almost two-thirds of WSW have benefited from STI screening during the last 12 months in different structures: associations, primary care centers, and rarely at their attending physician. The first intervention should be providing systematic information about PEP and PrEP when performing STI screening, whatever the sexual risk behaviors assessment. Indeed, WSW do not report their occupation to healthcare providers even when it would be appropriate to do so, and often underestimated the sexual health risks associated with their occupation.^[7,8]^ Fostering the awareness of sexual risk behaviors is essential. While most of the participants reported consistent condom use, nearly a third of the respondents reported being forced to have sex without a condom. Previous studies also acknowledge high rates of condom coercion.^[7–9]^ The appropriateness of PrEP versus PEP should be considered and discussed.

Second intervention could be the integration of PrEP into family planning services. Indeed, more than one-third of cisgender WSW interrogated had had an abortion. A previous study conducted in France found a similar high proportion of abortion among WSW, reaching 59%.^[10]^ Two recently published studies have explored the perspectives of women who have migrated from sub-Saharan Africa to France and of healthcare providers regarding PrEP implementation among migrant women attending family planning centers.^[11,12]^ Despite strong interest of migrant women for PrEP, both women and healthcare providers stressed the need for disseminating PrEP information and the challenge of adherence and follow-up. Healthcare providers underlined implementation challenges, including insufficient training and limited resources. Similar barriers to PrEP implementation have been notified by medical and social workers interviewed about PrEP use by migrant WSW.^[8]^

Finally, our study showed a high proportion of WSW with a family doctor who could play a determinant role in addressing their sexual healthcare needs. A larger study previously conducted in the South of France found that less than one-third of migrant WSW have a family doctor.^[10]^ Despite these discordant results regarding the proportion of WSW with family doctors, we demonstrated similar obstacles to access to care, including stigma and language barriers. In both studies, <30% of WSW had told their family doctor about their engagement in sex work. In Canada, where access to a general practitioner was very common among WSW, reaching >85%, the need for care adapted to the traumas and specificities of individuals engaging in sex work was also emphasized.^[13]^ The implementation of a national sexual health strategy in France, the edition of specific guidelines for addressing sexual health with migrants, and the recent extension of the authorization of PrEP prescription to general practitioners could favor the role played by family doctors in sexual healthcare.^[14,15]^ A recent survey conducted in Northern France found 72% of general practitioners were aware of PrEP.^[16]^ But the respondents stated that they rarely or never have seen individuals engaging in sex work and migrants in consultation.

Our study has several limitations. First, the numbers were low, particularly for transgender WSW. There appeared to be a difference in PrEP awareness between cisgender and transgender WSW. A study should be specific to transgender WSW. Second, this sample comprised only WSW attending the meetings of the association “Le Mouvement du Nid” in Lille, who may not be the most precarious. Moreover, only WSW who were fluent in spoken French, English, or Spanish and had a literacy level high enough to understand the nonopposition note were included. Finally, our study relies on self-report data. It may be subject to social desirability bias, underreporting of stigmatized issues, and overreporting of positive behaviors.

In conclusion, our study showed the lack of knowledge of HIV prevention tools among WSW, especially PrEP. Enhancement of sexual healthcare for WSW among family doctors may help PrEP implementation in this difficult-to-reach population.

## Author contributions

**Conceptualization:** Jérémie Robinsohn, Agnès Meybeck, Macha Tetart, Michel Valette.

**Data curation:** Jérémie Robinsohn.

**Formal analysis:** Jérémie Robinsohn, Agnès Meybeck, Emmanuelle Bontemps, Macha Tetart, Michel Valette.

**Methodology:** Agnès Meybeck, Michel Valette.

**Supervision:** Agnès Meybeck, Olivier Robineau.

**Validation:** Emmanuelle Bontemps, Macha Tetart, Olivier Robineau.

**Writing – original draft:** Jérémie Robinsohn, Agnès Meybeck.

**Writing – review & editing:** Jérémie Robinsohn, Agnès Meybeck, Emmanuelle Bontemps, Macha Tetart, Michel Valette, Olivier Robineau.


