# Supplementary material for: Assessment of healthcare access and knowledge of HIV pre-exposure prophylaxis in women engaging in sex work: An exploratory study in Lille, Northern France
Source: Medicine (Baltimore). 2026 Jul 24;105(30):e49609. doi: 10.1097/MD.0000000000049609 (PMC13406181; doi:10.1097/MD.0000000000049609)
Supplement: Supplementary file 1 [file medi-105-e49609-s001.docx]

Nom : coroPREP RIPH_2020_10

Prénom : N°IDRCB 2020-A02623-36

N°idencaon du parcipant : I__I__I__I

Date du quesonnaire : I__I__I I__I__I I__I__I__I__I

Questionnaire s’adressant aux personnes en situation de prostitution de la MEL visant

à évaluer les besoins et les connaissances de la PrEP comme prévention contre

l’infection par le VIH

Nom : coroPREP RIPH_2020_10

Prénom : N°IDRCB 2020-A02623-36

N°idencaon du parcipant : I__I__I__I

Date du quesonnaire : I__I__I I__I__I I__I__I__I__I

Questionnaire s’adressant aux personnes en situation de prostitution de la MEL visant

à évaluer les besoins et les connaissances de la PrEP comme prévention contre

l’infection par

***Questionnaire for Sex Workers in the Lille Metropolitan Area (MEL) to assess needs and knowledge regarding PrEP as HIV prevention***

**Part 1 : Who are you?**

1/ Are you ?

- Male
- Female
- Transgender person
- Other: please specify **__________**

2/ How old are you ?

- I am_______ years old

3 / Were you born in France ?

- Yes
- No, country of birth : _______

4/ If you answered « No » : at what age did you arrive in France ?

- 0 to 15 years
- 15 to 30 years
- 30 to 45 years
- 45 to 60 years
- > 60 years

5/ How long have you been engaged in sex work?

- ______

6/ How do you practice your activity (Multiple answers possible) ?

- independently
- As part of a network
- Escorting
- Through the internet

7/ Do you have children ?

- No
- 1 child
- 2 or more children

8/ Do you have social support ?

- I live with my partner
- I have family in the region
- I have friends in the region
- I do not have any relatives or close contacts in the region

9/ What is your housing situation ?

- I rent my home
- I own my home
- I live with my partner
- I live with my parents/or family
- I live with a friend
- Other. Please specify : _______

10/ What is your highest level of education ?

- I never attended school
- Primary school
- Secondary school
- High school graduate
- University

**Part 2 : global health**

11/ Do you have health insurance coverage ?

- I have supplementary health insurance
- I have Complementary Universal Health Coverage (C2S)
- I have State Medical Aid (AME)
- I do not have health insurance coverage

12/ Do you currently use the following (Multiple answers possible) ?

- Tobacco
- Cannabis
- Cocaine / heroin
- Alcohol
- Anxiolytics
- Antidepressants
- Sleeping pills

13/ How would you rate your overall health :

- Very good
- Good
- Fair
- Poor

**Part 3 : sexual practices**

14/ Do you have :

- Homosexual relationships
- Heterosexual relationships
- Bisexual relationships

15/ Number of clients per day :

- Between 0 and 2 clients per day
- Between 2 and 4 clients per day
- Between 5 et 10 clients per day
- More than 10 clients per day

16/ Is a condom used during penetrative sex :

- Always
- Almost always
- Often
- Almost never
- Never

17/ Is a condom used during oral sex :

- Always
- Almost always
- Often
- Almost never
- Never

18/ Do you ever agree to have sex without a condom in exchange for more money ?

- Never
- Sometimes
- Often
- Always

19/ During the past 12 months, have you experienced violence ? :

- No
- Yes

19.A/ If yes, what type(s) ?

- Theft / robbery
- Insults, or verbal abuse :
  - If yes, specify : from other sex workers ___ from a client ___ from a passer-by ___ from the police ___ from partners or relatives
- Physical violence
- Sexual violence

19.B/ Has a client ever forced you not to use codom ?

- Yes
- No

20/ After unprotected sex or condom breakage/removal, have you ever taken HIV post-exposure prophylaxis (PEP) ?

- Yes : specify
  - At a sexual health clinic (CEGIDD)
  - In an emergency department
  - At a medical clinic or with your general practitioner
  - other : specify________________
- No : specify
  - I am HIV-positive
  - I do not think I was at risk
  - I did not know about PEP
  - I did not know how to access it
  - I am not interested
  - Other : specify _________________

**Part 4 : Risk prevention**

21/ Do you have a chronic medical condition ?

- Hepatitis
- HIV/AIDS
- Diabetes
- Chronic respiratory disease
- Hypertension/dyslipidemia
- Psychological or psychiatric condition
- Other
- No chronic health condition

22/ Are you using contraception ?

- Yes : specify
  - Intrauterine device (IUD)
  - Hormonal implant
  - Oral contraceptive pill
- No

22/ Have you ever had a voluntary termination of pregnancy (abortion) ?

- Yes
- No

23/ Do you have a primary care physician (general practitioner) ?

- Yes
- No

24/ If yes, is your physician aware of your professional activity ?

- Yes
- No

25/ During the past 12 months, have you consulted a physician ?

- No
- Yes : specify (multiple answers possible)
  - General practitioner
  - At a sexual health clinic (CeGIDD)
  - Emergency department
  - At a community healthcare center (Médecins solidarité Lille (MSL) / ABEJ)
  - Gynecologist
  - Other : Specify ________

26/ Regarding sexually tranmitted infections (STIs), would you consider yourself :

- Well informed
- Poorly informed
- Not informed

27/ During the past 12 months, have you undergone STI screening ?

- Yes : specify where (multiple aswers possible)
  - General practitioner
  - At a sexual health clinic (CeGIDD)
  - Emergency department
  - At a community healthcare center (Médecins solidarité Lille (MSL) / ABEJ/ Entr’act)
  - Gynecologist
  - Other : Specify ________
- No

28/ If yes, did the screenin identify a STI ?

- yes
- No

29/ During the past 12 months, have you forgone medical care or screening?

- Yes
- No

29.A/ If yes, for wath reason?

- Financial reasons
- Language barrier
- I did not know where to go
- I was afraid of being asked overly personal questions
- Restrictive opening hours
- Fear of a hostile attitude from staff
- Fear of being asked for my papers/documents

30/ Have you ever heard of PrEP as HIV prevention ?

- No
- Yes, I am currently taking PrEP
- Yes , but I am not taking it
  - If you are not tataking PrEP, why not ?

(Multiple answers possible)

- - - I am HIV-positive
    - I do not thinkI need it
    - I am afraid of side effects
    - I do not think I can afford it
    - I am afraid I will forget to take it
    - I do not want to ask my doctor for it
    - Other : __________

30.A/ If you are taking PrEP, who initiated the treatment :

- Your general practitioner
- At a sexual health clinic (CeGIDD)
- At a community healthcare center (Médecins solidarité Lille (MSL) / ABEJ/ Entr’act)
- Other : __________

30.B/ If you are not familiar with PrEP :

*Information sheet and explanation about PrEP*

30.C/ After reading this information sheet, would you be interested in PrEP?

- Yes
- No
- I don’t know

31/ After completing this questionnaire would you like to benefit from : ?

- STI screening
- A medical consultation
- Neither
